# Species Composition, Abundance, and Habitat Association of Medium- and Large-Sized Mammals in and Around Yegof National Forest Priority Area, Wollo, Ethiopia

**DOI:** 10.1155/tswj/7937738

**Published:** 2025-08-13

**Authors:** Yohans Ayalew, Gezahegn Getachew, Dereje Yazezew

**Affiliations:** ^1^Department of Biology, College of Natural Sciences, Wollo University, Dessie, Ethiopia; ^2^Department of Biology, College of Natural and Computational Sciences, Debre Berhan University, Debre Berhan, Ethiopia

**Keywords:** abundance, biodiversity, mammals, primate, Yegof National Forest Priority Area

## Abstract

Understanding the composition, abundance, and habitat associations of mammalian species is crucial for their effective conservation and habitat management. The study was aimed at filling the knowledge gaps regarding mammalian diversity and distribution in the Yegof National Forest Priority Area and its surrounding habitats. Accordingly, we assessed the species composition, abundance, and habitat association of mammals in the study area from July 2021 to April 2022. Based on plant physiognomy, the study area was categorized into five habitat types: natural forest, wooded grassland, plantation, bushland, and open grassland. A total of 13 mammalian species were recorded through line transect surveys. The order Primate was the most abundant taxonomic group accounting for 68.9% of the total mammals recorded. Among the recorded species, *Papio anubis* was the most numerous, accounting for 32.77% of the total, followed by *Chlorocebus aethiops* (19.95%) and *Theropithecus gelada* (16.15%). The abundance of mammals varied significantly across different habitats. During the dry and wet seasons, 57.7% and 42.3% were recorded, respectively. A statistically significant seasonal difference in abundance was observed. More than half (56%) of the species recorded were common to all five habitat types. The highest species similarity index (SI = 0.95) was observed between natural forest and wooded grassland. The study reveals considerable mammalian diversity in the study area, accompanied by notable variations in abundance across different habitats and seasons. However, the study area faces conservation challenges, including deforestation, illegal hunting, and human–wildlife conflict, which threaten population sustainability. Addressing these threats is essential to ensure the long-term survival of mammal species.

## 1. Introduction

Mammals play crucial roles in terrestrial ecosystems, providing essential ecological services such as pollination, seed dispersal, and predation [1, 2]. They exhibit considerable diversity in both structure and function [3]. The class Mammalia globally includes 6495 species, of which 96 are extinct and 6399 remain extant [4]. East Africa is renowned for its rich diversity of mammalian fauna, with over 360 species recorded in the region [5]. Ethiopia alone is home to more than 320 mammalian species [3] and is recognized as one of the world's biodiversity hotspots, marked by a high degree of endemism [6]. This makes Ethiopia a global leader in both the richness and endemism of mammalian species [7]. Among the 320 mammalian species found in Ethiopia, 55 are endemic, a level of endemism surpassing that of many other African nations [6]. Furthermore, more than 60% of Ethiopia's mammal species are of medium to large size [8].

Globally, mammalian species encounter various threats [9], which are particularly significant for emerging wildlife protected areas. Large mammals, in particular, are more susceptible to local extinction than smaller species due to their extensive home-range requirements, naturally low population densities, and their tendency to be prime targets for hunting [10]. Evaluating the diversity and abundance of wildlife resources is a key aspect of conservation efforts [11]. As such, understanding the diversity, abundance, and habitat preferences of mammals is essential for determining their conservation status and formulating appropriate protection strategies [12–14].

Several studies have been conducted on the diversity of mammals in various protected areas of Ethiopia. However, a comprehensive inventory of mammals across the country's different ecosystem types is still lacking and remains poorly documented [7]. Yegof National Forest Priority Area (YNFPA), one of Ethiopia's youngest protected areas, has an underexplored faunal record. While research has focused on the ecology of small mammals, particularly rodent abundance and distribution in the area [15], there has been no prior investigation into the diversity and abundance of mammals. Thus, this study is aimed at (1) identifying the species composition of mammals in and around the YNFPA, (2) estimating the relative abundance of mammalian species across different habitat types, and (3) examining seasonal variation in mammal diversity and abundance between the dry and wet seasons.

The study hypothesizes that (1) species composition and abundance of mammal species are expected to be higher during the wet season than during the dry season. (2) Mammalian diversity and abundance are hypothesized to be greater in densely vegetated habitats with minimal human disturbance. (3) Different habitat types support significantly different mammalian species compositions and abundance, reflecting habitat-specific preferences or ecological requirements.

## 2. Materials and Methods

### 2.1. Description of the Study Area

**Figure 1:**
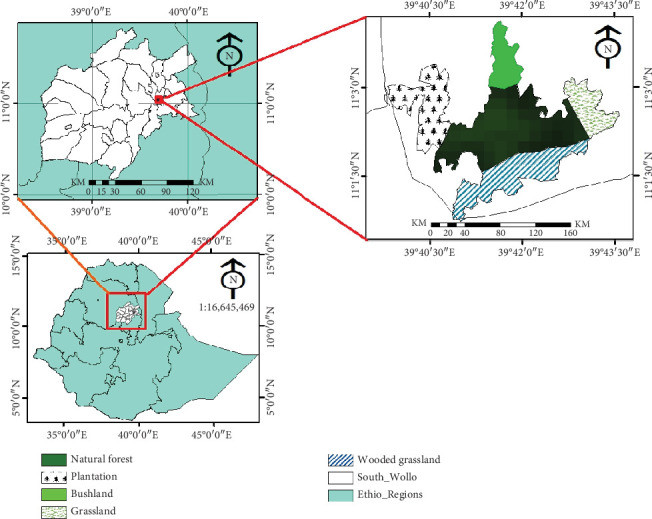
Map of Yegof National Forest Priority Area (*Source:* Goudar (2022), unpublished).

The study was conducted in the YNFPA, located in the South Wollo Zone of the Amhara Regional State, Ethiopia. The forest spans between latitudes 11°01⁣′ and 11°03⁣′ and longitudes 39°40⁣′–39°44⁣′, with an elevation ranging from 2000 to 3014 m above sea level [16–18]. It is situated 380 km north of Addis Ababa, 26 km southeast of Dessie, and 8 km west of Kombolcha. The study area is bordered by Kallu, Kombolcha, Albuko, and Dessie Zuria woredas and covers 1462.56 ha [16, 17] (Figure 1).

**Figure 2:**
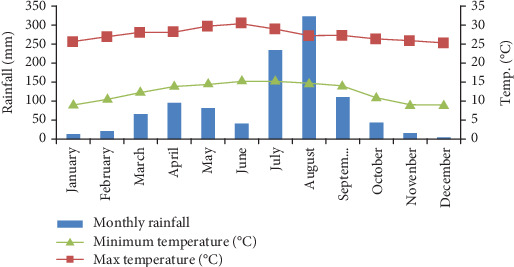
Monthly rainfall and maximum and minimum temperatures of the study area (2011–2020).

Based on data from the nearest weather station in Kombolcha Town, the area experiences a mean annual minimum temperature of 9.07°C and a maximum of 30.57°C, with an average annual temperature of 27.2°C (Figure 2). The study area experiences a bimodal rainfall pattern, with an average annual precipitation of 1041.8 mm. The primary rainy season occurs from June to September, while a shorter rainy period takes place between February and May, with intermittent rainfall [17, 18] (Figure 2). Higher rainfall and cooler temperatures are observed in the mountainous, forested regions due to their elevated altitude [18].

The Yegof Forest consists of natural highland vegetation, including dry evergreen, mixed conifer, and broadleaved trees, along with plantations of fast-growing exotic species. A total of 123 vascular plant species have been documented in the forest, representing 109 genera and 63 families. Among the dominant species are *Juniperus procera* and *Olea europaea* subsp. *cuspidata* [16, 18].

### 2.2. Sampling Design

A preliminary survey was conducted in the first week of July 2021 over 5 days, prior to the actual data collection. During this survey, data on climate, vegetation cover, topography, infrastructure, fauna, and other relevant factors were gathered. Based on the vegetation types and topography of the area, the study site was categorized into five habitat types, namely, natural forest, wooded grassland, plantation, bushland, and grassland. The line transect method, widely recognized for its effectiveness and cost-efficiency in surveying mammals in tropical forests and savannas, was employed in this study [19]. This method is effective for estimating the abundance of large and easily observable mammals [20]. The transect widths varied from 50 to 400 m, depending on the vegetation cover and topography of the area [13, 21–23]. To avoid double counting, transect widths were set at 100 m for natural forest; 200 m for plantation, wooded grassland, and bushland; and 400 m for grassland [24]. Depending on the topography and size of the habitat type, transect lengths ranged from 0.6 to 2 km [22, 23]. Data collection followed a stratified systematic sampling design. In total, 18 transect lines were established across the five habitat types: seven in natural forest, six in wooded grassland, two in plantation, two in bushland, and one in grassland. The number, length, and width of transects in each habitat type were adjusted based on vegetation density, topography, and habitat size [11].

### 2.3. Data Collection

Data on the composition of mammalian species were collected during both the dry (December to February) and wet (July to September) seasons, from July 2021 to April 2022, following the methods outlined in [25]. Each transect was visited three times per season [24]. Researchers walked quietly and gently along selected transects, moving against the wind to prevent human scent from reaching the mammalian species and reduce disturbances, as animals are particularly sensitive to odors carried by the wind. To assess mammal diversity, both direct observations and indirect evidence, such as footprints, body parts, vocalizations, burrows, spines, and droppings, were recorded [20]. Indirect evidence is particularly valuable when surveying animals that are rare, elusive, found at low densities, or difficult to observe repeatedly. Surveys were conducted twice a day during periods of peak animal activity: in the morning from 06:00 to 10:00 h and in the late afternoon from 16:00 to 18:00 h [26] in each transect. During each transect survey, data were recorded whenever an individual mammal, a group, or signs of mammals were observed. This included the habitat type, species name, the number of individuals per species, and GPS location [15]. Species identification was based on standardized field guides [27] and personal experience.

### 2.4. Data Analysis

The data collected were analyzed using SPSS software, Version 25. Both descriptive and inferential statistical methods were employed to present the results. The Shannon–Wiener diversity index was utilized to assess and quantify the diversity, abundance, and distribution of species across the different habitat types and between the wet and dry seasons using the formula outlined as follows [28]:

 $$\begin{array}{c} H^{\prime }=-\overset{n}{\underset{i}{\sum }}\left(\text{Pi}\right)\left(\text{lnPi}\right), \end{array}$$

where *H*′ is the diversity index, ln is the natural logarithm, and Pi is the proportion of total sample belonging to the *i*^th^ species (pi = ni/*N*, where ni is the number of individuals in species *i* and *N* is the total number of individuals of all species).

We also employed the Shannon–Wiener evenness index (*E*) to examine the distribution pattern of mammalian species across the habitats. This index was calculated using the following formula: *E* = *H*′/*H*_max_, where *H*′ represents the Shannon–Wiener diversity index and *H*_max_ is the maximum possible diversity, calculated as lnS. In this formula, ln denotes the natural logarithm and *S* refers to the total number of species present in each habitat.

Sorenson's coefficient (*S*) was used to assess the similarity in mammalian species composition across different habitat types using the following formula: *S* = 2*C*/(*S*1 + *S*2 + *S*3+⋯Sn) [28], where *C* represents the number of species common to all habitat types, *S*1 is the number of species in habitat one, *S*2 is the number of species in habitat two, *S*3 is the number of species in habitat three, and Sn is the number of species in *n*^th^ habitat. To evaluate the relative abundance index (RAI) of each species, we calculated the proportion of records for each species relative to the total number of records for all species. The relative abundance was computed as follows: Relative abundance = (*n*/*N*)∗100, where *n* is the number of individuals of a specific species recorded and *N* is the total number of individuals of all species in the study area. We employed the chi-square test to examine whether there were significant associations between mammal species composition and abundance across different habitats and seasons, with a significance level set at *p* ≤ 0.05.

## 3. Results

### 3.1. Species Composition of Mammals

In this study, a total of 421 individuals of mammals belonging to 13 species were recorded. These species belonged to six different orders and nine families (Table 1). Species richness varied across the different orders and families. Among the orders, Carnivora was the most diverse, representing 30.9% of the species (four species), followed by Artiodactyla and Primates, each contributing 23% of the species (three species) in the study area.

### 3.2. Abundance of Mammals

**Figure 3:**
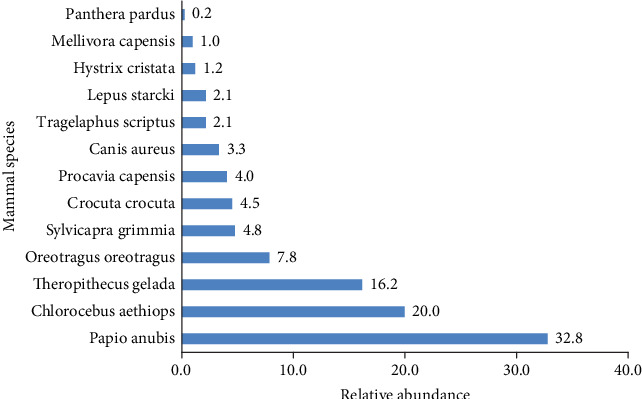
The relative abundance of medium- and large-sized mammals in YNFPA.

The order Primates was the most abundant (68.9%), followed by Artiodactyla (14.7%). The least abundant order was Rodentia, which accounted for only five individuals. Among species, *Papio anubis* was the most abundant, comprising 32.8% (138 individuals), followed by *Chlorocebus aethiops* with 20% (84 individuals). *Panthera pardus* was the least abundant species, making up only 0.2% relative abundance (one individual) of the total in the study area (Figure 3 and Table 2).

### 3.3. Habitat Association of Mammals

The highest number of individual mammal species was recorded in the natural forest (11 species), followed by the wooded grassland (10 species) (Table 2). The species richness across the different habitats in the study area did not exhibit statistically significant differences (*χ*^2^ = 1.682, df = 4, *p* > 0.05). However, the abundance of mammals varied significantly between habitats (*χ*^2^ = 96.328, df = 4, *p* < 0.05).

### 3.4. Seasonal Variation of Mammals

Of the total mammals recorded, 57.7% (243 individuals) were observed during the dry season, while 42.3% (178 individuals) were recorded during the wet season (Table 2). The seasonal variation in mammal abundance in the study area was statistically significant (*χ*^2^ = 10.04, df = 1, *p* < 0.05). Among the recorded mammal species, only *P. anubis* showed a significant seasonal variation (*χ*^2^ = 4.9, df = 1, *p* < 0.05), with a higher abundance observed during the dry season (Table 2).

Among the different habitat types, a significant difference in the abundance of mammals was observed in the natural forests between the wet and dry seasons (*χ*^2^ = 11.77, df = 1, *p* < 0.05), with distinct variations in their numbers between the two seasons.

### 3.5. Diversity and Evenness of Mammals Among Habitats

The natural forest has the highest species diversity (*H*′ = 1.99), followed by the wooded grassland (*H*′ = 1.94), and the least was recorded in grassland (*H*′ = 1.26). Regarding species evenness, the plantation habitat had the highest evenness (*J* = 0.96), followed by the wooded grassland (*J* = 0.84), while the grassland had the lowest evenness (*J* = 0.60) (Table 3).

### 3.6. Species Similarity Between Habitats

The highest species similarity (SI = 0.95) was recorded between the natural forest and wooded grassland followed by the grassland and bushland habitats (SI = 0.94) in the study area (Table 4).

## 4. Discussion

In the present study, a total of 13 species of mammals were recorded in and around YNFPA. This finding is consistent with the results of [29], who also reported 13 mammal species in Guda Forest, Southwestern Ethiopia. The similarity between these two findings can likely be attributed to comparable climatic conditions, vegetation cover, and conservation status of both areas. However, the total number of mammal species recorded in YNFPA is relatively lower compared to other regions [14, 22, 23, 30, 31]. There are a number of factors that may explain the lower species diversity found in this study. These are the relatively small size of the YNFPA, human disturbance levels, habitat heterogeneity, and conservation management intensity.

The small area limits the occurrence of microhabitats and ecological niches, thus restricting the presence of habitat specialists and wide-ranging species [32]. In addition, intensive human activities like agricultural expansion, fuelwood extraction, and illegal hunting impact wildlife populations, both on their abundance and diversity [33]. Relative to the Humbo Community-Based Forest Area in Southern Ethiopia, with just eight mammal species [21], the Yegof area had comparatively higher species richness. This may be due to differences in habitat type, successional stage, and topographic complexity [34]. In contrast, Yegof has moist evergreen montane forest with old-growth vegetation and a more broken landscape, which could provide greater habitat complexity and refugia from human disturbance. Topographic heterogeneity, specifically, has been implicated in higher species richness by contributing microhabitat diversity and escape terrain for fauna [35].

Consistent with previous research carried out in the Ethiopian highlands (e.g., Dati Wolel National Park [15]; Wabe forest fragments; Gurage Zone [26]; Harego Forest, South Wollo [13]; and Wacha Protected Forest in Western Ethiopia [25]), the order Primates was most represented in Yegof forest, followed by Artiodactyla. This could be an indication of the ecological flexibility of primates, particularly *P. anubis* and *C. aethiops*, to adapt to habitat fragmentation and human proximity more than other mammalian orders [36]. They exhibit diversified foraging strategies and are adapted to consume a wide range of food items. Additionally, factors such as their ability to thrive in varied climatic and topographic conditions, high reproductive success, adaptability to different habitats, and their higher tolerance to human disturbances may also contribute to their abundance in these areas [9].

The abundance of mammals was higher during the dry season, a finding contrary to the results reported by [22] in the Adaba Community Forest, Ethiopia [23]; Southern Great Rift Valley, Ethiopia; and [21] in the Humbo Community-Based Forest Area. The increased abundance of mammals during the dry season may be due to the fact that more people enter the forest during the rainy season to collect firewood and harvest grass for livestock, leading to higher human disturbance. This encroachment by humans and livestock limits the foraging opportunities for mammals and makes their detection and monitoring more challenging. Studies from various regions have shown that livestock encroachment and human settlements negatively affect the abundance and distribution of wild mammals, which reduces the chances of sighting these species [37]. Additionally, during the wet season, the growth of herbaceous and ground vegetation may provide thick cover, making it harder to spot mammals. Heavy rain and mist during the wet season further contribute to poor visibility of these species. Similar effects have been observed in Kainji Lake National Park, Nigeria, where excessive rainfall and vegetation outgrowth hindered the observation of vervet monkeys [38].

Among the habitat types, only natural forests exhibited significant variation in mammal abundance between the wet and dry seasons. This indicates that the mammal populations in the natural forests are affected by the seasonal changes. Natural forests supported a higher abundance of mammal species during the dry season, similar to the findings of [39] in Mengaza communal forest, East Gojjam, Ethiopia. Particularly, *P. anubis* was the most abundant species during the dry season in both study sites. This could be attributed to mammals moving from peripheral areas and other habitat types toward the natural forest during the dry season for shelter. The natural forest provided a water source and palatable grasses, making it a key refuge for mammals during this time. Other possible reasons for the increased abundance of mammals in natural forests during the dry season could be thermal regulation with denser canopy cover, reduced human disturbance, and better cover and concealment from predators.

The present study revealed variation in species diversity among different habitats, with natural forests exhibiting the highest species diversity, while grasslands showed the lowest. This finding aligns with studies conducted in other regions of Ethiopia, such as in the Wacha Protected Forest, Western Ethiopia [26] and Guda Forest, Southwestern Ethiopia [29]. The variation in species diversity may be attributed to differences in habitat quality and the specific habitat preferences of the species.

## 5. Conclusions

This study investigated the species composition, abundance, and habitat association of medium- and large-sized mammals in and around YNFPA. A total of 13 mammal species were recorded, with *P. anubis* (olive baboon) being the most abundant species and Primates the most represented order, followed by Artiodactyla. Species diversity varied across habitat types, with the highest diversity observed in natural forests and the lowest in grasslands. Seasonal differences were also evident, with mammal abundance being significantly higher during the dry season, particularly in natural forests. This pattern may be attributed to reduced human disturbance, better visibility, and greater access to essential resources such as food, water, and shelter during the dry season. Notably, natural forests showed a marked increase in species abundance during the dry season compared to other habitat types, suggesting their critical role as dry season refugia. These findings highlight the ecological importance of natural forests for supporting mammalian diversity and indicate that habitat type and seasonality are key factors influencing mammal distribution and abundance in the YNFPA landscape. Therefore, strengthening conservation efforts and expanding habitat protection are essential for the conservation of the mammalian diversity in the area and ensuring the long-term survival of these species and enhancing ecosystem resilience.

## Figures and Tables

**Table 1:** List of mammal species identified in YNFPA.

**Order**	**Family**	**Scientific name**	**Common name**	**Evidences**
Artiodactyla	Bovidae	*Oreotragus oreotragus*	Klipspringer	Visual
		*Tragelaphus scriptus*	Common bushbuck	Visual
		*Sylvicapra grimmia*	Common duiker	Visual

Carnivora	Hyaenidae	*Crocuta crocuta*	Spotted hyena	Visual
	Felidae	*Panthera pardus*	Leopard	Sound/scat/local report
	Mustelidae	*Mellivora capensis*	Honey badger	Visual
	Canidae	*Canis aureus*	Common jackal	Visual

Rodentia	Hystricidae	*Hystrix cristata*	Porcupine	Visual/spine

Hyracoidea	Procaviidae	*Procavia capensis*	Rock hyrax	Visual

Lagomorpha	Leporidae	*Lepus starcki*	Stark's hare	Visual

Primates	Cercopithecidae	*Papio anubis*	Olive baboon	Visual
		*Theropithecus gelada*	Gelada	Visual
		*Chlorocebus aethiops*	Grivet monkey	Visual/scat

**Table 2:** Mammalian species abundance in different habitats of YNFPA between seasons.

**Species scientific name**	**Habitat type/season**										**Total**
	**Natural forest**		**Wooded grassland**		**Grassland**		**Plantation**		**Bushland**		
	**Wet**	**Dry**	**Wet**	**Dry**	**Wet**	**Dry**	**Wet**	**Dry**	**Wet**	**Dry**	
*Oreotragus oreotragus*	5	7	6	6	1	2	2	2	1	1	33
*Tragelaphus scriptus*	1	3	2	3	—	—	—	—	—	—	9
*Sylvicapra grimmia*	3	6	4	5	—	—	—	—	—	2	20
*Crocuta crocuta*	3	3	2	3	1	1	1	1	2	2	19
*Mellivora capensis*	—	—	—	—	1	—	1	—	1	1	4
*Hystrix cristata*	—	—	—	—	1	—	1	1	1	1	5
*Theropithecus gelada*	7	15	11	12	3	2	4	4	5	5	68
*Procavia capensis*	5	6	2	4	—	—	—	—	—	—	17
*Lepus starcki*	2	3	2	2	—	—	—	—	—	—	9
*Canis aureus*	2	3	1	2	1	1	1	1	1	1	14
*Chlorocebus aethiops*	9	16	11	13	10	14	—	—	5	6	84
*Papio anubis*	11	25	13	18	19	16	—	6	13	17	138
*Panthera pardus*	—	1	—	—	—	—	—	—	—	—	1
Total no. of spp.	10	11	10	10	8	7	6	6	8	9	13
Relative abundance (%) per season	11.4	21	12.8	16.2	8.8	8.5	2.4	3.5	6.9	8.5	100
Relative abundance (%) per habitat	32.3		29		17.3		6		15.4		100

**Table 3:** Diversity index and evenness of mammals in different habitats.

**Habitat type**	**No specie (** **S** **)**	**Total abundance**	**Shannon–Weaver index (** **H**∗**)**	**H**_max_ = **Ln (S)**	**Evenness **(**J**) = **H**/**H**_max_
Natural forest	11	136	1.99	2.39	0.83
Wooded grassland	10	122	1.94	2.30	0.84
Grassland	8	73	1.26	2.08	0.60
Plantation	6	25	1.72	1.79	0.96
Bushland	9	65	1.60	2.19	0.73
Total	13	421			

**Table 4:** Species similarity in different habitats in the study area.

**Habitat**	**Natural forest**	**Wooded grassland**	**Grassland**	**Plantation**	**Bushland**
Natural forest	1				
Wooded grassland	0.95	1			
Grassland	0.63	0.67	1		
Plantation	0.56	0.59	0.93	1	
Bushland	0.7	0.74	0.94	0.88	1

## Data Availability

The data used for this study are available from the corresponding author upon reasonable request.
